# Beyond Risk Factors: Rethinking Hepatitis B and Hepatitis C Screening in Primary Care

**DOI:** 10.1111/liv.70330

**Published:** 2025-09-19

**Authors:** Elena Vargas‐Accarino, Ariadna Rando‐Segura, María Asunción Úbeda, Imma Valls, Carla Ventosa, Núria García, Ingrid Arcusa, Elena Monserrate, Eva de Diego, Marta Selvi, Adriana Palom, Marina Llorens, Maria Santos, Judit Romero‐Vico, Artur Dalfo, Juan Carlos Ruiz‐Cobo, Ester Pallares, María Buti

**Affiliations:** ^1^ Medicine Department Universitat Autònoma de Barcelona Barcelona Spain; ^2^ Liver Diseases Research Group, Vall d'Hebron Research Institute Barcelona Spain; ^3^ CIBERehd enfermedades hepáticas y digestivas Madrid Spain; ^4^ Microbiology Department Vall d'Hebron University Hospital Barcelona Spain; ^5^ CAP Horta Barcelona Spain; ^6^ CAP San Rafael Barcelona Spain; ^7^ CAP Guineueta Barcelona Spain; ^8^ CAP Río de Janeiro Barcelona Spain; ^9^ CAP Trinitat Vella Barcelona Spain; ^10^ CAP Chafarinas Barcelona Spain; ^11^ CAP Sant Andreu Barcelona Spain; ^12^ Liver Unit Vall d'Hebron University Hospital Barcelona Spain

**Keywords:** hepatitis B, hepatitis C, primary care, screening

## Abstract

**Introduction:**

Most international guidelines recommend screening for hepatitis B virus (HBV) and hepatitis C virus (HCV) only for individuals with risk factors or elevated ALT levels. However, this approach may not suffice to eradicate viral hepatitis. This study evaluates the application of EASL HBV and HCV screening guidelines in primary care centres (PCCs).

**Methods:**

The study included two components: (1) A retrospective review (January 2021–March 2023) of microbiology data to determine testing rates, analyse clinical characteristics, and assess management; and (2) A prospective intervention (March–April 2024) involving HBV and HCV screening and risk factor surveys for all adults attending two PCCs for blood collection.

**Results:**

In the retrospective analysis of 90 170 patients, HBV and HCV screening rates were 16% and 10%, respectively. Among HBsAg‐positive patients (*n* = 84 (0.5%)), 67% lacked risk factors or elevated ALT. Among anti‐HCV‐positive (*n* = 277 (3%)) and HCV RNA‐positive (*n* = 45 (0.5%)) patients, 54% and 46% respectively lacked these indicators.

In the prospective study of 1030 patients (mean age 55, 39.6% men, 73% Spanish), anti‐HCV was detected in 1.16% of cases (HCV RNA in 0.19%). Of these, 67% lacked risk factors or elevated ALT. No HBsAg‐positive cases were identified, and hepatitis B vaccination status was uncertain for 50% of patients.

**Conclusion:**

Risk factor‐based screening for HBV and HCV in primary care is a suboptimal approach. More than half of the patients testing positive lacked identifiable risk factors or elevated ALT levels. Universal one‐time screening for all adults could address these limitations and significantly advance viral hepatitis elimination efforts.

**Abbreviations**
Anti‐HCV: hepatitis C antibodies
APRI: AST‐Platelet ratio index
CDC: Centers of Disease Control and Prevention
EASL: European Association for the Study of Liver
HBsAg: hepatitis B surface antigen
HBV: hepatitis B virus
HCV: hepatitis C virus
PCC: primary care centers
WHO: world health organization

**Summary**

- Less than 20% of adults attending primary care centers were screened for hepatitis B or C. More than half the patients with these infections had no risk factors or elevated ALT levels. Based on these screening criteria, HBV and HCV testing may not have been performed.
- In the prospective study, the prevalence of HCV was twice as high in patients with no risk factors or elevated ALT.
- Viral hepatitis screening for adults in primary care centres independent of risk factors could contribute to achieving the WHO goals of viral hepatitis elimination by 2030.

## 1 Introduction

Chronic hepatitis C virus (HCV) and hepatitis B virus (HBV) infections pose significant public health challenges, contributing to substantial global morbidity and mortality [1]. The long‐term consequences of chronic viral hepatitis include severe liver diseases, such as cirrhosis and hepatocellular carcinoma, which collectively cause 1.34 million deaths per year. Notably, HCV and HBV are specifically implicated in half of all liver cancer cases, making them the third leading cause of cancer‐related deaths worldwide [2].

In response to this critical situation, the World Health Organization (WHO) has set a goal to reduce hepatitis‐related deaths by 65% and new infections by 90% by 2030 [3, 4]. Fortunately, highly effective treatments are available for these infections. For HCV, direct‐acting antivirals (DAAs) attain cure rates exceeding 95% [5], while nucleosid(t)ide analogues for HBV achieve viral suppression in more than 90% of treated individuals [6]. Therapeutic drugs, together with robust diagnostic tests, are the main tools to achieve the WHO's hepatitis elimination goal [6, 7, 8].

In high‐income countries, various strategies have been implemented to enhance the detection of HCV and HBV infections [9, 10, 11, 12]. Traditionally, screening has been based on risk factors, but a more recent approach involves universal screening for all adults once in their lifetime. The 2020 recommendations from the US Centers for Disease Control and Prevention (CDC) [13] advocate for universal, one‐time hepatitis screening for all individuals aged 18 years and older. This update expands upon previous recommendations focused on risk‐based screening approaches and is expected to increase the identification and treatment of hepatitis cases [13].

Nonetheless, most international guidelines, including those from the European Association for the Study of Liver (EASL) [7] and Spanish health authorities [14], recommend screening for viral hepatitis only in individuals with risk factors or elevated aminotransferase levels. These recommendations are supported by descriptive studies showing that over 80% of patients with hepatitis B and C exhibit identifiable risk factors for infection [14, 15]. For example, a study conducted by the Spanish Ministry of Health in 2019 estimated the prevalence of antibodies against HCV at 0.85%, with the prevalence of HCV RNA and hepatitis B surface antigen (HBsAg) both at 0.22% [14].

Primary care physicians, as the first point of contact in the Spanish public health system, play a crucial role in detecting individuals with viral hepatitis. In addition, they are responsible for referring them to appropriate centres for further evaluation and treatment. In Spain, HCV and HBV therapy is given exclusively through hospital pharmacies, and efficient specialist referral is essential to ensure timely access to care.

This study aimed to evaluate real‐world viral hepatitis detection and management in a primary care setting. Two assessments were conducted. The first was a retrospective database review to determine HCV and HBV testing rates, analyse clinical characteristics of patients testing positive, and evaluate management in daily practice. The second was a prospective intervention, in which patients attending PCCs and undergoing blood collection were offered HCV and HBV testing and administered an epidemiologic survey to investigate the appropriateness of current hepatitis screening guidelines.

## 2 Patients and Methods

This multicenter study consists of two parts. The first involved a retrospective search in the microbiology database of an urban health area in Barcelona (Spain), encompassing 7 primary care centres (PCCs). The study researchers identified all blood samples tested for HCV (anti‐HCV antibodies) or HBV (HBsAg) between January 2021 and March 2023. For samples testing positive, reflex testing for HCV RNA and HBV DNA was performed, respectively. The medical records of patients with positive anti‐HCV or HBsAg results were then reviewed by 2 or 3 study collaborators to identify risk factors for viral hepatitis and transaminase elevations, and to determine the rates of patients linked to care or lost to follow‐up.

The second part of the study, conducted at 2 primary care centers between March and April 2024, was a prospective exploratory intervention that included HCV and HBV determinations as well as a patient survey administered at the same time as the blood draw. The rationale for the survey was to ensure structured collection of risk factors and other relevant viral hepatitis‐related information, including HBV vaccination status (Appendix S1). All patients undergoing blood testing for any reason at these PCCs during a 1‐month period, who provided oral consent to participate, were included. Testing involved determination of HBsAg and hepatitis C antibodies (anti‐HCV) in all participating patients. Reflex testing for HBV DNA and anti‐HDV was conducted for those with positive HBsAg results, while HCV RNA was analysed for patients with positive anti‐HCV findings. All individuals with positive viral hepatitis results were linked to appropriate care.

### 2.1 Statistics

Categorical variables were compared using the chi‐square test or the Fisher exact test when frequencies were less than 5%, and are expressed as frequency and percentage. Quantitative variables were analysed with the Mann–Whitney *U* test or Student *t* test, as appropriate, and are expressed as mean and standard deviation. Results were considered statistically significant at *p*‐values lower than 0.05. All statistical analyses were carried out using GraphPad Prism 6.

### 2.2 Ethical Considerations

All data were processed confidentially in a database accessible only to the researchers, in keeping with Spanish legislation. Informed consent for participation in the retrospective part was waived with the approval of the hospital ethics committee because no interventions other than those of regular clinical practice were carried out. Oral consent for participation was given in the prospective part. This study was approved by the Research Ethics Committee of Vall d'Hebron Hospital (PR(AG)162/2024), and was conducted following good clinical practice guidelines.

## 3 Results

### 3.1 Retrospective Study

**FIGURE 1:**
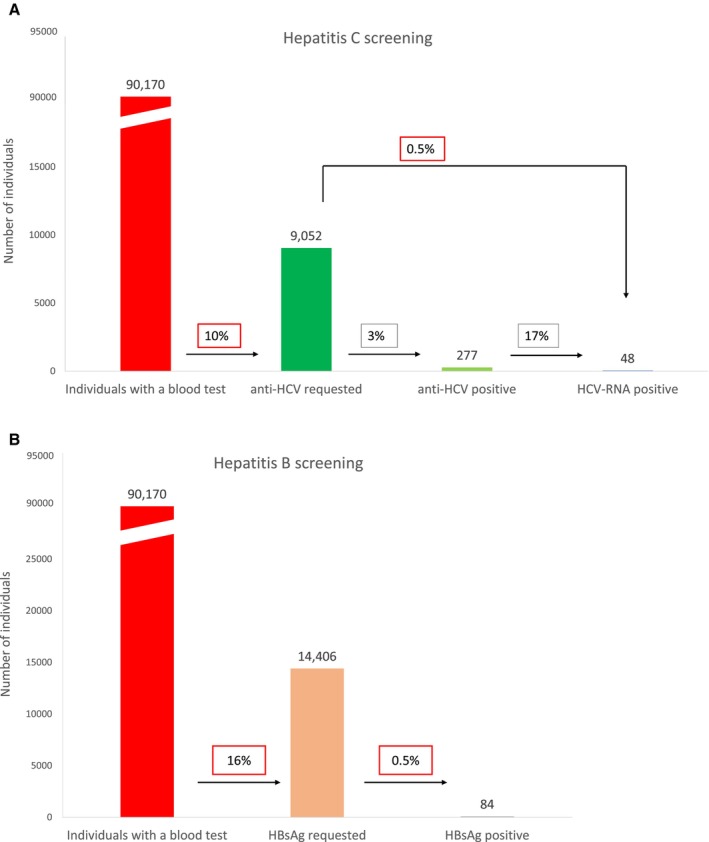
Flow chart of the study results. (A) Hepatitis C screening. (B) Hepatitis B screening.

In the first part of the study, 90 170 serum samples from consecutive adults attending 7 PCCs were reviewed between January 2021 and March 2023. For patients with more than 1 sample during this period, only the first was included. Primary care physicians had requested anti‐HCV antibody testing in 9052 samples (10%), of which 277 (3%) were anti‐HCV‐positive and 48 (0.5%) had detectable HCV RNA (Figure 1A). HBsAg testing had been requested in 14 406 (16%) samples, with 84 (0.5%) testing positive (Figure 1B).

The demographic and clinical characteristics of patients with positive results for anti‐HCV, HCV RNA, and HBsAg were analysed and compared. Anti‐HCV‐positive individuals were predominantly men, native Spaniards, with 24% showing elevated ALT levels, and a low prevalence of significant fibrosis, as indicated by noninvasive markers. Patients positive for HCV RNA were significantly older, had higher ALT levels, and more frequently demonstrated significant fibrosis based on FIB‐4 results than either anti‐HCV‐positive or HBsAg‐positive patients.

Among 277 anti‐HCV‐positive patients, 65 (24%) had elevated ALT levels and 107 (38.6%) had at least one risk factor. Reported risk factors included intravenous drug use in 52 (19%) patients, a history of blood transfusion in 26 (9%), tattoos in 12 (4%), sexual risk behaviours in 12 (4%), and vertical transmission in 5 (2%). Risk factors plus elevated ALT levels were documented in 127 (45.8%) patients. Notably, 170 (62%) patients had no risk factors, 202 (76%) had ALT levels within normal limits, and 150 (54%) patients had neither risk factors nor abnormal ALT levels. Among the 48 patients testing positive for HCV RNA, 42% reported risk factors, 31% had elevated transaminases, and 46% exhibited neither of these parameters.

Forty‐two (87.5%) of the 48 HCV RNA‐positive patients were followed. Of these, 28 (58%) started DAAs, including 11 (40%) patients with fibrosis stage F3–F4 and one with decompensated liver disease. Fourteen (29%) additional patients were not treatment candidates due to a limited life expectancy or severe comorbidities, and 6 (13%) moved to another region and were lost to follow‐up.

The 84 HBsAg‐positive patients were significantly younger and included a higher percentage of migrants than those testing positive to HCV (Table 1). Among the HBsAg‐positive samples, 5 (5.9%) were HBeAg positive, 72 (85.7%) had detectable HBV DNA (median level, 487.5 ± 20 192 341 IU/mL), and 5 (5.9%) were anti‐HDV positive, including 1 with detectable HDV RNA.

**TABLE 1:** Demographic and clinical characteristics of anti‐HCV+, HCV RNA+, and HBsAg+ patients in the retrospective part of the study.

Parameters	Anti‐HCV+ (*n* = 277)	HCV RNA+ (*n* = 48)	HBsAg+ (*n* = 84)	*p*
Sex, male	152 (55%)	29 (60%)	46 (54.8%)	0.77
Age, years	57.2 ± 15.4	68.4 ± 22.2	42 ± 17.5	< 0.0001****
Born in Spain	195 (70%)	34 (71%)	26 (30.9%)	< 0.0001****
Risk factors	107 (38.6%)	20 (42%)	11 (13.1%)	< 0.0001****
ALT (IU/L)	25.8 ± 19.9	46.8 ± 39.5	25 ± 51.9	< 0.0001****
Elevated ALT	65 (24%)	15 (31%)	19 (22.6%)	0.47
No risk nor ALT > 40 IU/L	150 (54%)	22 (46%)	56 (66.7%)	0.043*
FIB‐4, median	2.26 ± 11.6	2.1 ± 1.36	1.28 ± 1.13	< 0.0001****
FIB‐4 > 3.25	11 (4%)	8 (17%)	2 (2.4%)	0.0005***
APRI, median	0.36 ± 0.54	0.6 ± 0.42	0.47 ± 0.54	< 0.0001****
APRI > 1.5	3 (1%)	2 (4%)	3 (3.6%)	0.097

*Note:* Categorical variables are expressed as *n* (%) and quantitative as mean ± SD.

Abbreviation: APRI, AST‐Platelet Ratio Index.

*p ≤ 0.05, **p ≤ 0.01, ***p ≤ 0.001, ****p ≤ 0.0001.

Regarding the recommended screening criteria in relation to HBV, 19 (22.6%) of the 84 patients testing positive had elevated ALT levels and 11 (13.1%) reported at least 1 risk factor, including sexual risk behaviour (4 patients), tattoos (1), previous blood transfusion (3), and vertical transmission (3). In total, 56 (66.7%) patients exhibited neither hepatitis risk factors nor elevated ALT levels.

Among the 4 anti‐HDV‐positive patients, 25% were Caucasian, with a mean age of 39.7 years. Only one had detectable HDV RNA, and one had elevated ALT levels.

Of the 90 170 analyses performed, 7767 (8.6%) serum samples had ALT levels higher than the upper limit of normal. HCV screening was conducted on 1948 (25%) of these samples and HBsAg screening on 2002 (25.7%), with 1703 samples screened for both anti‐HCV and HBsAg. Among the total screened, anti‐HCV antibodies were detected in 65 (3.33%) samples and HBsAg was identified in 19 (0.9%).

Seventy‐seven (92%) of the 84 HBsAg‐positive patients were followed: 12 had chronic hepatitis (5 HBeAg positive and 7 HBeAg negative) and 65 had chronic HBV infection.

### 3.2 Prospective Study

**FIGURE 2:**
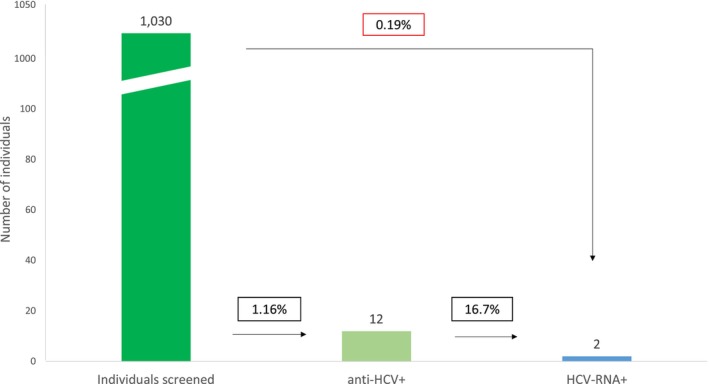
Flowchart of the prospective hepatitis C screening.

In the prospective part of the study, conducted between March and April 2024, HCV and HBV screening was performed and an epidemiological survey was administered to consenting adults attending two PCCs for blood collection for any reason. Among a total of 1213 consecutive individuals, 1030 (84.9%) agreed to participate: 408 (39.6%) men, median age 57 ± 19.3 years, and 755 (73.3%) born in Spain. ALT levels were elevated in 95 (9.22%) patients, and 343 (33.3%) reported risk factors, including tattoos (*n* = 177), sexual risk behaviour (*n* = 71), prior blood transfusion (*N* = 66), possible vertical transmission (*n* = 28), and intravenous drug use (*n* = 1). Anti‐HCV antibodies were detected in 12 (1.16%) patients. Three of them were aware of the infection and reported that they had been treated and cured. HCV RNA was detected in 2 of the 12 anti‐HCV‐positive samples (0.19%). Both these patients started HCV treatment (Figure 2).

Anti‐HCV antibodies were identified in 2 (0.7%) of the 304 individuals with risk factors and in 2 (3.6%) of the 56 patients with elevated ALT levels. None of the 39 individuals exhibiting both risk factors and elevated ALT levels were positive for anti‐HCV. However, 8 (1.35%) of the 631 individuals without risk factors and with normal ALT levels tested positive.

HBsAg was not detected in any sample of the 1030 patients screened, even though 343 patients reported risk factors and 95 had elevated ALT levels. However, 13 patients reported a previous history of viral hepatitis. The survey found the following regarding HBV vaccination: 511 (49.7%) patients were unsure whether they had been vaccinated, 261 (25.3%) reported having received HBV vaccine, and 258 (25%) had not been vaccinated. Among this last group, there was a significantly higher percentage of individuals born outside of Spain than native Spanish patients (33.7% vs. 21.7%) (*p* = 0.002).

## 4 Discussion

Within the public health care setting, primary care physicians have a significant role in early detection of viral hepatitis, by ordering diagnostic blood tests. Current European and Spanish guidelines recommend viral hepatitis screening for individuals with risk factors or clinical signs of the disease [6, 7]. In the retrospective part of the present study, we investigated HCV and HBV screening rates and linkage to care in PCCs. Among all individuals undergoing blood collection in 7 PCCs within the study period, 10% were tested for anti‐HCV and 16% for HBsAg. Being retrospective data, it was not possible to determine whether the prompts for these test requests conformed to the guideline recommendations. As the relevant data were not systematically collected, patients may have underreported risk factors due to reluctance, the stigma related to these factors, and other potential barriers [16]. In addition, certain high‐risk viral hepatitis populations, such as people who inject drugs and migrant groups, may be attended to and managed in available public health centers other than PCCs [17, 18].

However, we were able to investigate risk factors and elevated ALT levels among the subset of patients with positive viral testing. Notably, in 54% of patients who tested positive for anti‐HCV and in 66.7% of those positive for HBsAg, the records showed no risk factors and ALT levels were within normal limits. This is concerning because, under our current guideline recommendations, these patients would not have been tested. These findings suggest that relying solely on risk factors and ALT elevations for HCV and HBV screening may hinder accurate and timely diagnoses. This notion is supported by the results of 2 studies performed in Spanish emergency departments, where 60% of HCV‐positive and 92% of HBV‐positive patients reported no risk factors for viral hepatitis [16, 19].

In the case of elevated transaminases, the requirement for three separate medical visits in our setting—first for ALT determination, then to check if levels are elevated, and finally for HCV or HBV testing—may further complicate viral hepatitis identification and linkage to care [15, 20]. Our retrospective study found that 25% of patients with elevated ALT levels were tested for HBV or HCV. This rate is somewhat higher than the values reported in a retrospective study from Belgium that reviewed data from 89 PCCs [21]. HbsAg and anti‐HCV testing was conducted in only 17.5% and 11.8%, respectively, of patients with abnormal ALT levels. However, information on viral hepatitis risk factors was lacking in 60% of these patients, a situation that may have contributed to the lower testing rates.

Another important aspect to highlight is the higher percentage of individuals successfully linked to care in our project (87.5% for HCV and 92% for HBV patients) compared to other studies. For example, in a similar study conducted in the German primary care setting, the percentage of new HCV and HBV patients linked to care was 47% and 83%, respectively [22]. This higher linkage to care observed in our cohort may be attributed to several factors, including universal free access to treatment, which may reduce loss to follow‐up compared to a private healthcare system [23], and ongoing educational sessions provided to primary care professionals in our health area [24]. It is important to note that linkage‐to‐care rates in other regions of Spain are not as high as those observed in our cohort [25], suggesting that increasing awareness and training among primary care physicians could enhance viral hepatitis management.

The second part of our study was an exploratory prospective investigation, in which participants were consistently asked about risk factors using a structured questionnaire, and all were tested for viral hepatitis. The aim was to have a more reliable evaluation of the appropriateness of viral testing based on the guideline criteria. However, we were only able to assess testing in relation to hepatitis C, as none of the patients screened in the prospective study were positive for hepatitis B.

Anti‐HCV antibodies were detected in 1.16% of the patients studied, with 16.7% of them testing HCV RNA‐positive. These percentages are in line with the low prevalence of HCV reported in a primary care study by the Spanish Ministry of Health in 2020 [14]: anti‐HCV positivity was 0.85% with 26% of viremic cases. The somewhat lower percentage of active HCV infections in the present study may be related to the impact of DAA treatment, which has significantly reduced the number of active infections by achieving high cure rates in treated individuals [5].

In total, 399 patients (38% of the 1030 surveyed) reported risk factors for viral hepatitis or had elevated ALT levels. Among these, 4 patients tested positive for anti‐HCV antibodies. In the remaining 631 patients, who did not meet the guideline‐based recommendations, 8 additional HCV‐positive cases were detected. Of particular note, the prevalence of anti‐HCV antibodies was higher among patients without risk factors or elevated ALT levels (1.35%) than in those with risk factors or abnormal ALT (0.7%).

The related literature contains very few studies on HBV and HCV testing in primary care. A study similar to ours, performed in a non‐urban primary care facility in Flanders [26] analysed the prevalence of HCV infection and associated risk factors among adult patients undergoing a blood test. Over the course of 1 year, 560 people were screened, and anti‐HCV antibodies were detected in 5 (0.89%) cases, a percentage similar to the value found in our prospective cohort. Notably, 1 of the 5 HCV‐positive patients would not have been identified using targeted screening. The authors concluded by advocating for once‐in‐a‐lifetime population screening.

Our study has limitations. In the retrospective component, risk factors were extracted from clinical records, which may not ensure consistent documentation across all participants. In the exploratory prospective component, the limited sample size and study duration might explain the absence of HBV‐positive patients.

In conclusion, 2 key findings emerged from the study. In the retrospective analysis, risk factors were absent and ALT levels were within normal limits in more than half of HCV‐ or HBV‐infected patients, suggesting a significant shortcoming in the effectiveness of current risk‐based screening recommendations. In the prospective part, universal screening identified twice as many HCV‐positive patients in the group without risk factors or abnormal ALT compared to those with these characteristics. These results strongly suggest that existing HCV screening guidelines fail to detect a substantial proportion of infections and underscore the need to reconsider universal screening strategies in the effort to eliminate viral hepatitis.

## Author Contributions

Conceptualization, M.B.; methodology, M.B., A.R.‐S. and E.P.; software, E.V.‐A.; validation, E.V.‐A., and M.B.; formal analysis, E.V.‐A.; investigation, E.V.‐A., M.A.U., I.V., C.V., N.G., I.A., E.M., E.D., M.S., A.P., M.L., M.S., J.R.‐V., A.D., J.C.R.‐C.; resources, E.V.‐A.; data curation, E.V.‐A.; writing – original draft preparation, E.V.‐A.; writing – review and editing, E.V.‐A., M.B.; visualisation E.V.‐A., M.B.; supervision M.B.; project administration, M.B.; funding acquisition, M.B. All authors have read and agreed to the published version of the manuscript.

## Ethics Statement

This study was approved by the Research Ethics Committee of Vall d'Hebron Hospital (PR(AG)162/2024), and was conducted following good clinical practice guidelines.

## Consent

Informed consent for participation in the retrospective part was waived with approval of the hospital ethics committee because no interventions other than those of regular clinical practice were carried out. Oral consent for participation was given in the prospective part.

## Conflicts of Interest

M.B. has served as a speaker and advisory board member for Gilead, Roche, and Arbutus. J.C.R.‐C. has served as a speaker for Gilead.

## Supporting information




**Appendix S1:** liv70330‐sup‐0001‐AppendixS1.pdf.

## Data Availability

The data that support the findings of this study are available from the corresponding author upon reasonable request.
