# Supplementary material for: Beyond Risk Factors: Rethinking Hepatitis B and Hepatitis C Screening in Primary Care
Source: Liver Int. 2025 Sep 19;45(10):e70330. doi: 10.1111/liv.70330 (PMC12447784; doi:10.1111/liv.70330)
Supplement: Supplementary file 1 — Appendix S1: liv70330‐sup‐0001‐AppendixS1.pdf. [file LIV-45-0-s001.pdf]

HEPATITIS SCREENING SURVEY

Name and surname: Subject ID:

Date of birth: Age:

DEMOGRAPHIC DATA

Sex: ☐ Male ☐ Female

Country of birth: ____________________________________

How long have you been living in Spain? _________________________

CLINICAL DATA

Are you aware of being a hepatitis C carrier? ☐ Yes ☐ No

If yes, in what year were you diagnosed? ________________

If yes, do you undergo regular follow-up with a specialist doctor? ☐ Yes ☐ No

Are you vaccinated against hepatitis B? ☐ Yes ☐ No

Are you aware of being a hepatitis B carrier? ☐ Yes ☐ No

If yes, in what year were you diagnosed? ________________

If yes, do you undergo regular follow-up with a specialist doctor ? ☐ Yes ☐ No

Do any of the following apply to you?

- ☐ You have received a blood transfusion
- ☐ You have tattoos or piercings
- ☐ You have had scalp micropigmentation
- ☐ You have used injectable drugs
- ☐ You are a man and have had sex with other men
- ☐ Your mother is a hepatitis B carrier
